# Traditional Plant-Derived Compounds Inhibit Cell Migration and Induce Novel Cytoskeletal Effects in Glioblastoma Cells

**DOI:** 10.3390/jox14020036

**Published:** 2024-05-09

**Authors:** Evan Thompson, Sally Prior, Anke Brüning-Richardson

**Affiliations:** School of Applied Sciences, University of Huddersfield, Huddersfield HD1 3DH, UK

**Keywords:** glioblastoma, migration, anti-migratory, invasion, 2D/3D assays, turmeric, indigo, magnolia

## Abstract

Glioblastomas (GBMs) are aggressive and invasive cancers of the brain, associated with high rates of tumour recurrence and poor patient outcomes despite initial treatment. Targeting cell migration is therefore of interest in highly invasive cancers such as GBMs, to prevent tumour dissemination and regrowth. One current aim of GBM research focuses on assessing the anti-migratory properties of novel or repurposed inhibitors, including plant-based drugs which display anti-cancer properties. We investigated the potential anti-migratory activity of plant-based products with known cytotoxic effects in cancers, using a range of two-dimensional (2D) and three-dimensional (3D) migration and invasion assays as well as immunofluorescence microscopy to determine the specific anti-migratory and phenotypic effects of three plant-derived compounds, Turmeric, Indigo and Magnolia bark, on established glioma cell lines. Migrastatic activity was observed in all three drugs, with Turmeric exerting the most inhibitory effect on GBM cell migration into scratches and from the spheroid edge at all the timepoints investigated (*p* < 0.001). We also observed novel cytoskeletal phenotypes affecting actin and the focal adhesion dynamics. As our in vitro results determined that Turmeric, Indigo and Magnolia are promising migrastatic drugs, we suggest additional experimentation at the whole organism level to further validate these novel findings.

## 1. Introduction

Glioblastoma (GBM) is a form of brain tumour that is particularly problematic due to its aggressive nature and high proliferative capacity, promoting tumour recurrence in around 90% of patients [1]. GBM is classified as a grade IV astrocytoma, meaning that it arises from astrocytes of the brain, most commonly in the temporal and frontal lobes [2]. GBMs are widely accepted to be one of the most morbid cancers, with only 40% surviving their first year of diagnosis, and as little as 17% surviving their second. In untreated cases, GBMs can cause patient death within six months. The standard treatment of GBMs involves surgical removal or resection, followed by a combination of chemotherapy and radiotherapy, though this has only proven effective to moderately prolong life expectancy and is not curative [3].

The poor survival rate associated with GBMs is directly linked to their location and propensity to invade healthy brain parenchyma. Complete tumor excision reduces patient mortality; however, this is rarely possible due to the lack of a distinct border between the GBM and normal brain tissue [4]. Research aiming to improve GBM outcomes has resulted in several novel treatments such as inhibitor therapy, immunotherapy and drug-delivering nanoparticles, but these have failed to translate into the clinic as they provide no significant survival benefit when compared to standard treatments [5,6]. Tumor recurrence is inevitable due to the invasive nature of GBM cells, a characteristic which fails to be targeted by current GBM therapeutics. Cancer cells migrate by hijacking the molecular machinery of the cell, utilizing the cytoskeleton and adhesion proteins for motility [7]. GBMs adopt mesenchymal migration, characterized by an elongated cell body and lamellipodia at the cells’ leading edge to drive migration [8]. Both actin stress fibers and focal adhesions (FAs) generate the force and adhesion required for migration [9]. As the migratory capacity of GBM cells aids tumor recurrence, it is therefore of interest to target migration in highly invasive cancers such as GBMs, to promote patient survival.

Drugs produced from natural sources are invaluable in the field of drug discovery and development, as they can provide complex compounds without requiring synthesis in a laboratory. Natural compounds are highly diverse, offering a plethora of chemical structures to test, potentially leading to the discovery of novel mechanisms of action to combat conditions where therapeutic progress has remained unchanged. It is estimated that around 80% of humans rely on plant-based drugs as there are so many available on the market [10]. Within the field of cancer research, approximately 50% of internationally approved anti-cancer drugs have arisen from natural sources [11].

**Figure 1:**
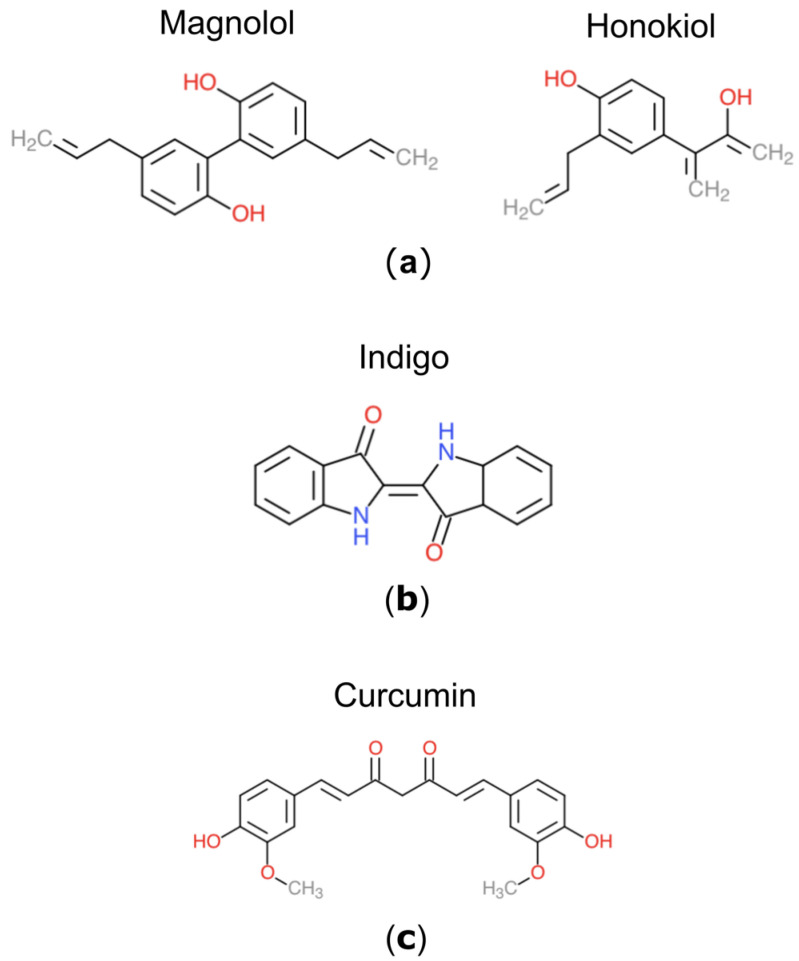
The structure of three plant-derived compounds. (**a**) Magnolia possesses two APIs, Magnolol and Honokiol. (**b**) Indigo is derived from the Indigofera tinctoria plant. (**c**) Curcumin is the API found in Turmeric.

Three plant-derived compounds which have recently gained interest for anti-cancer properties are Turmeric, Magnolia Bark and Indigo (Figure 1). Magnolia bark, derived from the Magnolia tree, is multifunctional due to the presence of two active pharmacological ingredients (APIs), Magnolol and Honokiol [12]. Various properties of Magnolia Bark have been determined, such as anti-inflammatory, antimicrobial, antioxidant, neuroprotective and cytotoxic activity [13]. Honokiol has been suggested to regulate cell signalling, proliferation and growth inhibition in various tumors, but has also shown efficacy as an adjuvant agent alongside standard chemotherapeutics and radiotherapy to overcome drug resistance [14,15]. Animal and in vitro studies of Magnolol have also shown promise in reducing tumor growth [16]. Turmeric is widely used for a range of health benefits due to its API, Curcumin, which has been shown to be therapeutically viable. Curcumin is present at low concentrations (3%) within Turmeric, where it exhibits antioxidant and anti-inflammatory properties, but also appears to play a role in the prevention of heart disease, Alzheimer’s, and cancer [17]. Various studies have demonstrated the ability of Curcumin to induce apoptosis, whilst preventing angiogenesis, metastasis and growth [18,19]. Similar to Magnolia Bark, Curcumin has also shown efficacy in generating DNA damage to induce apoptosis in the established LN229 glioma cell line when administered as an adjuvant alongside EGFR kinase inhibitors [20,21]. Derived from the Indigofera tinctoria plant, Indigo is a natural dye used cosmetically to improve hair and scalp quality and reduce hair loss [22]. Indigo has been observed to induce apoptosis in patients with acute promyelocytic leukaemia, although poor solubility was noted [23]. Several APIs are found in Indigo, such as indirubins, which are a promising group of compounds, including BIO-indirubin (BIO), which appear to exert anti-migratory effects on glioma cells [24]. BIO has demonstrated efficient targeting of cell migration in paediatric gliomas, whilst also acting as a cytotoxic potentiator of cell death in adult GBM [25].

Although many plant-based compounds have been investigated for their potential cytotoxic and anti-cancer effects, the migrastatic activity of these compounds has so far not been evaluated, particularly in GBMs. Here we describe anti-migratory activity and novel phenotypic effects of three plant-derived candidate drugs to target cell migration including, Magnolia bark, Indirubin and Curcumin. We aimed to assess the anti-migratory activity of these compounds, with the hope of identifying novel treatment options to increase the efficacy of chemotherapy and radiotherapy, by limiting GBM cell migration to ultimately improve patient outcomes. Utilizing 2D phenotypic and scratch assays, 3D spheroid invasion assays and immunofluorescence microscopy enabled the identification and quantification of the effects that Turmeric, Indigo and Magnolia bark have on cell migration and invasion. This study highlights the potential use of plant-derived therapeutics as novel treatment options for GBMs, alongside current treatment regimens, to promote GBM patient survival. Therefore, this supports the wider use of naturally sourced compounds as therapeutic candidates.

## 2. Materials and Methods

### 2.1. Cell Culture

The GBM cell line, U87, obtained from ATCC (previously STR (short tandem repeat) profiled and mycoplasma tested), was cultured in complete medium, consisting of Dulbecco’s Modified Eagles Medium (DMEM) (Fisher Scientific, Leicester, UK), supplemented with 10% heat-inactivated fetal calf serum (FCS) (Sigma-Aldrich, Canterbury, UK) and 1% Penicillin/Streptomycin (Sigma-Aldrich, Canterbury, UK) in an incubator at 37 °C and 5% CO_2_ atmosphere.

### 2.2. Drug Preparation

Turmeric (New Leaf Products, Amazon, UK), Magnolia Bark (Nutrivity, Amazon, UK) and Indigo (It’s Pure Organics, Amazon, UK) were crushed into powders, weighed and resolubilized in dimethyl sulfoxide (DMSO) (Sigma-Aldrich, Canterbury, UK) to a working concentration of 100 mg/mL. The highest concentration of DMSO used in this study was in the higher magnolia concentration and was calculated as 0.116%, which is within the accepted DMSO level of 0.1 to 0.5% to prevent any effects on cell viability in *in vitro* assays.

### 2.3. MTT Assay

MTT ((3-(4,5-dimethylthiazol-2-yl)-2,5-diphenyltetrazolium bromide) assays were carried out as previously described [26]. Following trypsinization, U87 cells were seeded at a density of 1 × 10^4^ cells/mL in complete medium, before 200 µL of cell suspension was added to each well of columns 2 to 12, in flat-bottomed 96-well plates (Starlab, Milton Keynes, UK). A volume of 200 µL of complete media was added to each well of column 1, and plates were incubated for 24 h. Drugs were diluted to 100 µg/mL in complete media, before a two-fold range of dilutions was carried out in columns 3 to 12. Columns 1 and 2 contained complete media only and complete media containing 0.1% DMSO, to act as internal controls. Plates were then incubated for 96 h, and 20 µL of 5 mg/mL MTT (Sigma-Aldrich, Canterbury, UK) dissolved in phosphate buffered saline (PBS) (Sigma-Aldrich, Canterbury, UK) was added to each well. Media was removed after 4 h, 150 µL of DMSO (Fisher Scientific, Leicester, UK) was added, mixed thoroughly, and each plate was read on a FLUOstar Ultima Microplate Reader at 540 nm. From the readouts, dose-response curves were established, based on percentage cell survival, which allowed the determination of IC_25_ and IC_50_ values. The IC_25_ was calculated for each drug here as the concentration resulting in at least 75% cell viability, to ensure that the compounds were used at concentrations that target cell migration rather than proliferation. MTT assays were repeated in duplicate.

### 2.4. 2D Phenotypic Assay and Immunofluorescence

Coverslips (Sigma-Aldrich, Canterbury, UK) were immersed in 100% methanol (Sigma-Aldrich, Canterbury, UK) under sterile conditions and placed in 6-well plates (Starlab, Milton Keynes, UK) following methanol evaporation. The U87 cell suspension was diluted to 1 × 10^3^ cells/mL and seeded on top of the coverslips, then incubated for 4 h to induce cell attachment. Media was replaced with 2 mL of either control (0.1% DMSO) or drug treatment (0.027 µg/mL Indigo, 0.031 µg/mL Turmeric and 0.116 µg/mL Magnolia, 1:1000 dilutions of the IC_25_ values determined via MTT assays). Migratory activity was observed every 24 h on an EVOS XL Core imaging system (Fisher Scientific, Leicester, UK), as part of the migration/invasion assays. After adding 4% paraformaldehyde (PFA) (Sigma-Aldrich, Canterbury, UK), coverslips were incubated at room temperature for 15 min, washed 3 times in PBS and incubated for a further 5 min with 0.05% Triton X-100 (Sigma-Aldrich, Canterbury, UK) for permeabilization. The coverslips were washed with PBS 3 times, before 0.05% blocker was prepared from skimmed milk powder (ASDA, Leeds, UK) and PBS, and added to the wells. To observe cellular actin distributions and FA dynamics, DAPI (4′,6-diamidino-2-phenylindole) (EMD Millipore Corp, Danvers, MA, USA) was used to highlight the nucleus, TRITC (tetramethyl rhodamine) conjugated phalloidin (ECM Biosciences, Aurora, CO, USA) was used for actin localization and mouse anti-vinculin (EMD Millipore Corp, Danvers, MA, USA) was used to visualize FAs. Mouse anti-vinculin (1:500), DAPI and TRITC-conjugated phalloidin (both 1:1000) were diluted in the blocker solution and centrifuged for 5 min at 13,000 rpm. Drops measuring 200 µL each of primary antibody supernatant were transferred onto strips of Parafilm (Fisher Scientific, Leicester, UK) floating on water, before coverslips were placed cell side down in the antibody, covered in foil and incubated at room temperature for an hour. After being transferred back into the wells, coverslips were washed 3 times with PBS, whilst the secondary antibody was prepared as above to allow visualization of specific vinculin staining (Alexa Fluor 488 goat anti-mouse, 1:500, Abcam, Cambridge, UK). Antibody incubation was carried out as before. Two drops of MOWIOL (Sigma-Aldrich, Canterbury, UK) were added to each labelled glass slide (Sigma-Aldrich, Canterbury, UK), and coverslips were placed onto the slides cell side down, covered in tinfoil and allowed to airdry overnight at room temperature.

### 2.5. Scratch Assay

Cells were seeded at a concentration of 1 × 10^5^ cells/mL in 6-well plates (Starlab, Milton Keynes, UK) and incubated for 48 h. A 200 µL pipette tip was used to produce scratches horizontally across the center of each coverslip, and media was replaced with 2 mL of either control (0.1% DMSO) or drug treatment (27.02 µg/mL Indigo, 0.027 µg/mL Indigo, 31.94 µg/mL Turmeric, 0.032 µg/mL Turmeric, 116.07 µg/mL Magnolia and 0.116 µg/mL Magnolia). Concentrations for each compound determined by MTT to target cell migration rather than proliferation were used to inhibit cell migration, and cells were also treated with lower concentrations of the compounds to confirm compound-specific, subtle effects on the actin cytoskeleton and FA dynamics. Migration into the scratches was monitored at 0 and 24 h using an EVOS XL Core imaging system (Fisher Scientific, Leicester, UK), as we previously established that optimum migratory activity in U87 could be observed within 24 h of carrying out the assay.

ImageJ (v2.9.0) package Fiji (v1.53t) was used to calculate the area of each scratch, using the ‘polygon selection’ tool to outline the cell free scratch area [27]. Migration was quantified by calculating the scratch index reciprocal (SIR) as below:

$$\frac{1}{24\;hr\;Scratch\;Area\;÷0\;hr\;Scratch\;Area}.$$

### 2.6. Spheroid Invasion Assay and Immunofluorescence

Ultra-low adherence round-bottomed 96-well plates (Corning, Deeside, UK) were seeded with U87 cells at a concentration of 5 × 10^3^ cells/mL and incubated for 96 h to promote cell aggregation and spheroid formation. Spheroid invasion assays and subsequent analysis were carried out as previously described [25], with spheroids being treated with either control (0.1% DMSO) or drug treatment (27.02 µg/mL Indigo, 0.027 g/mL Indigo, 31.94 µg/mL Turmeric, 0.032 µg/mL Turmeric, 116.07 µg/mL Magnolia and 0.116 µg/mL Magnolia). Both high and low concentrations of the three drugs were used to inhibit cell migration and observe any subtle, specific cytoskeletal effects in the cells, respectively. Images were obtained of the spheroids in their collagen plugs every 24 h for a total of 48 h, using an EVOS XL Core imaging system (Fisher Scientific, Leicester, UK). The timeframe of the experiment over 48 h was chosen to allow maximum cell migration for this cell line to assess migratory activity, whilst preventing migration beyond the imaging field of view. Analysis of cell migration/invasion into a collagen matrix was quantified as previously described, by calculating the migration index (MI) for both the migrating front and migrating edge.

Following experimental completion, each collagen plug was washed 3 times with PBS and incubated with 4% PFA overnight, at room temperature, covered in tinfoil. Both primary and secondary antibody incubations were carried out as described for the 2D phenotypic assays; however, spheroids were stained in the 96-well plates. For mounting, the whole collagen plugs containing the migratory cells and spheroids were gently removed from the individual wells using a pastette and transferred into MOWIOL onto a glass slide. A coverslip was added, and the specimens were allowed to airdry overnight as before.

### 2.7. Confocal Microscopy

All slides were imaged using a Zeiss LSM 880, fitted with a Zeiss Plan-APOCHROMAT 63× and an EC Plan-Neofluar 10× objective lens. Alexa fluor 488 (secondary to mouse anti-vinculin) was excited at a wavelength of 488 nm, TRITC-conjugated phalloidin at 561 nm to highlight the actin cytoskeleton and DAPI at 405 nm to visualize the nucleus.

### 2.8. Focal Adhesion Analysis

To determine the effects of drug treatment on FA generation, images acquired on the Zeiss LSM 880 were opened in Fiji and split into their respective color channels [27]. FAs were observed via the use of Alexa fluor 488 as a secondary antibody to mouse anti-vinculin (as mentioned in Section 2.4.) to ascertain the location and number of FAs according to their distinct fluorescent signal. As previously reported for the monoclonal antibody, FAs appeared as bright distinct spots/focal contacts within the cells, which were easily recognizable for scoring. To score the number of FAs, the total number of both FAs and cells were counted using the green channel only, before the number of FAs per cell was calculated for each drug treatment and the control.

### 2.9. Actin Localization Analysis

Similarly to the FA analysis, images were split into their color channels, before the red channel was selected. Distinct actin localization categories were determined from all images and each cell was categorized accordingly into one of three groups (cortical, stress fibers or fragmented) and counted, before the percentage of cells in each category was calculated.

### 2.10. Statistical Analysis

All data analysis was carried out using IBM SPSS Statistics (v28) [28]. Kolmogorov-Smirnov tests were used to determine if data were normally distributed or not (*n* ≥ 50). An ANOVA was carried out followed by a Tukey post hoc test if data were normally distributed, whilst a Kruskal-Wallis followed by a post hoc test was carried out on non-normally distributed data.

## 3. Results

### 3.1. MTT Assays

IC_25_ values were determined for each of the plant-derived compounds. Turmeric had an IC_25_ of 31.94 µg/mL, Indigo was calculated as 27.02 µg/mL and Magnolia bark was 116.07 µg/mL. The concentrations were established to be anti-migratory rather than cytotoxic and therefore used in the assays. A MTT graph is shown in the Supplementary Materials section (Figure S1).

### 3.2. Two-Dimensional Phenotypic Assay

Images of the 2D assays acquired via confocal microscopy highlighted differences in cytoskeletal organization following treatment with the three plant-derived compounds.

#### 3.2.1. Actin Localization

**Figure 2:**
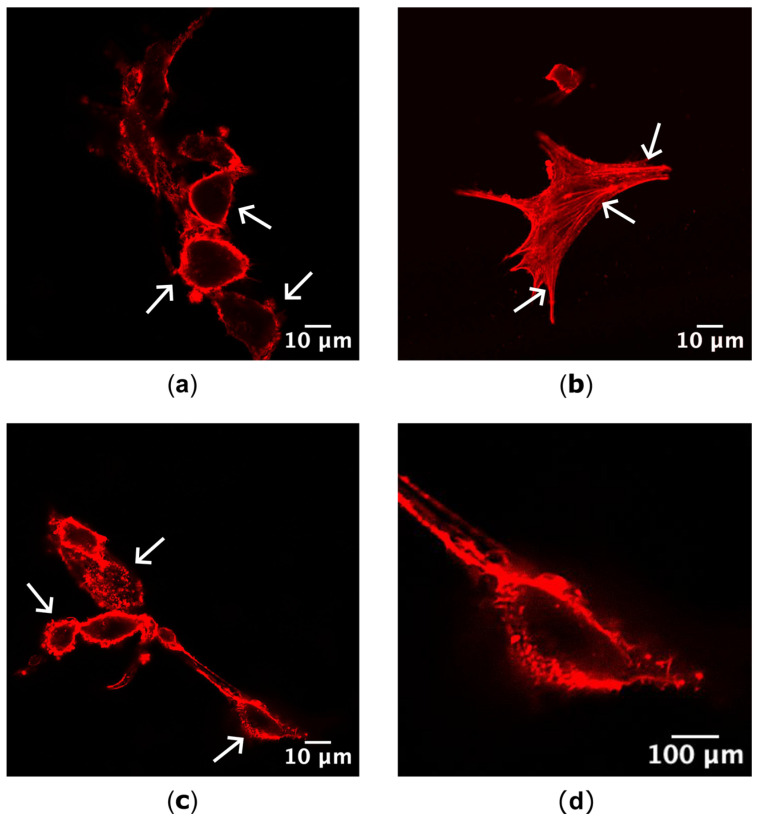
Actin localization categories identified via confocal microscopy. (**a**) Actin was localized cortically, around the perimeter of the cell, following treatment with Indigo (27.02 µg/mL). (**b**) Stress fiber formation across the cell body was observed in control, mock-treated cells. (**c**) Fragmented actin was located peripherally, but appeared to be formed from short, random actin filaments in Indigo (27.02 µg/mL) treated cells. (**d**) Magnified cell from (**c**) highlighting the fragmented actin phenotype. Arrows highlight examples for each actin category identified in cells. Red = actin; for (**a**–**c**) scale bar = 10 µm, for (**d**) scale bar = 100 µm.

Differences in actin localization as a result of drug treatment were visible and distinct, with cells possessing either cortical actin concentrated around the cells’ perimeter, stress fibers or actin fragments with a bleb-like appearance (Figure 2).

**Figure 3:**
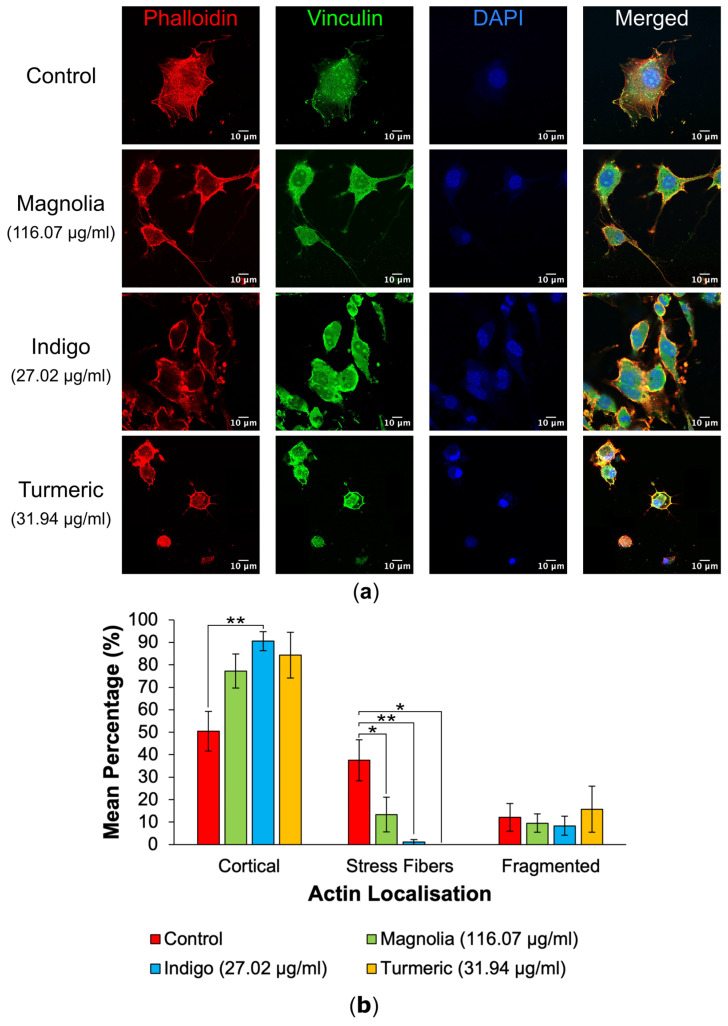
Immunofluorescence images and actin localization analysis of both mock-treated and treated U87 cells. (**a**) Images of control, Magnolia (116.07 µg/mL), Indigo (27.02 µg/mL) and Turmeric (31.94 µg/mL) treated cells were imaged, with Alexa fluor 488 (secondary to mouse anti-vinculin) excited at a wavelength of 488nm, TRITC-conjugated phalloidin at 561 nm and DAPI at 405 nm. The mock-treated control group is characterized by actin stress fibers, whereas Turmeric appears to induce a shift towards cortical actin localization concomitant with a rounded, amoeboid morphology. Scale bar = 10 µm. (**b**) Mean percentage of actin localizations (mean ± SEM). Indigo appeared to exert the most significant changes in actin dynamics. Red = actin, green = vinculin and blue = DAPI. *n* ≥ 7 for each drug treatment, based on two repeats. Kruskal-Wallis: * = *p* ≤ 0.05 and ** = *p* ≤ 0.01.

Actin localization in mock-treated cells versus drug-treated cells was quantified following visualization via confocal microscopy to highlight any potential effects of each plant-derived drug on cytoskeletal organization (Figure 3a). Control mock-treated cells mainly exhibited actin on their cortex, with all treatments possessing a higher mean percentage of cortical actin in comparison to the control; however, this was only significant for Indigo (27.02 µg/mL) (Figure 3b). Stress fiber formation was significantly reduced after all three drug treatments, and was the most significant for Indigo, despite treatment with Turmeric (31.94 µg/mL) seemingly resulting in total inhibition of actin stress fiber formation. The mean percentage of fragmented actin was the highest following treatment with Turmeric; however, this was determined to be statistically insignificant.

#### 3.2.2. FA Generation

**Figure 4:**
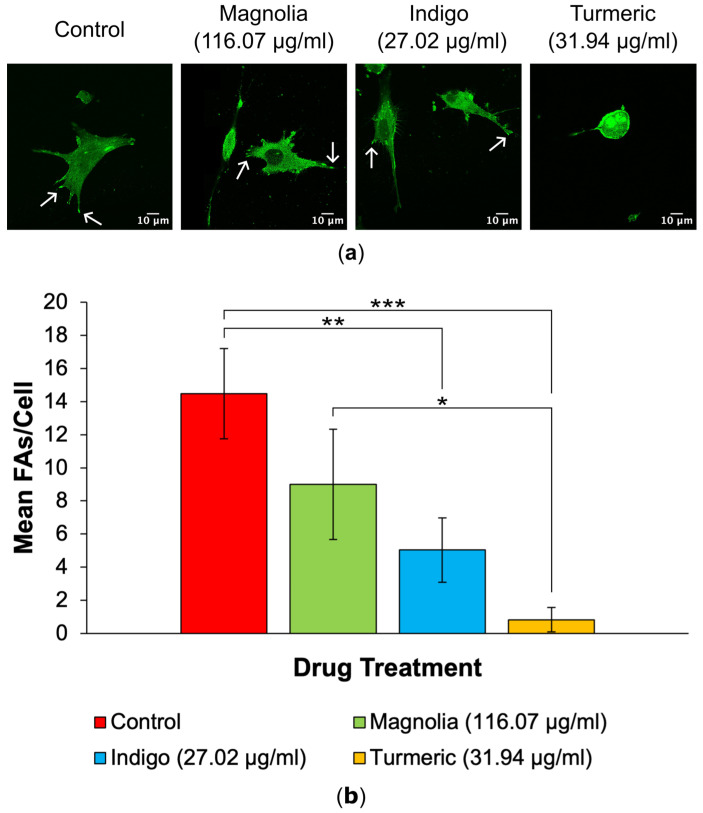
Plant-derived drugs reduce FA numbers in U87 cells. (**a**) Images generated via confocal microscopy show vinculin expression and highlight a decrease in FA numbers in all three plant-based compounds in comparison to the mock-treated control. Arrows show example FAs for each treatment group. Scale bar = 10 µm. (**b**) Mean number of FAs per cell for each drug treatment (mean ± SEM). Magnolia, Indigo and Turmeric all decreased the number of FAs per cell, with Turmeric exhibiting the most inhibitory effect on FA generation; *n* ≥ 4 for each drug treatment. Kruskal-Wallis: * = *p* ≤ 0.05, ** = *p* ≤ 0.01 and *** = *p* ≤ 0.001.

A decrease in FA numbers was observed in all drug treatments, with Turmeric (31.94 µg/mL) displaying the lowest mean number of FAs per cell, which appears to be directly correlated with the observed rounded, amoeboid morphology in Turmeric-treated cells (Figure 4a). Cells subjected to treatment with Indigo (27.02 µg/mL) also exhibited statistically significantly decreased FA numbers, but this was less significant than for Turmeric (Figure 4b).

### 3.3. Scratch Assays

#### 3.3.1. Drugs Administered at a Low Concentration

**Figure 5:**
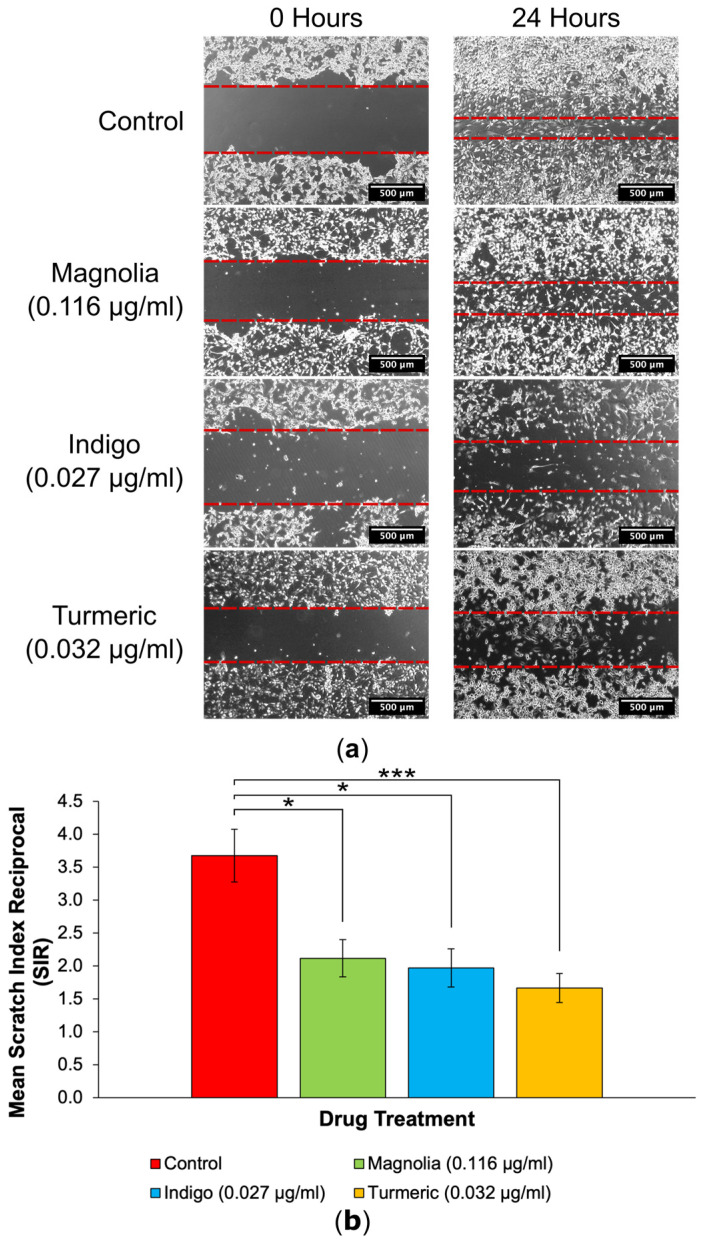
Two-dimensional migration into scratches is significantly reduced by plant-derived compounds with established anti-cancer activity at low concentrations. (**a**) EVOS XL Core generated images demonstrated the extent of migration into scratches after 24 h incubation. Dashed red lines are used to outline the wound edges. Scale bar = 500 µm. (**b**) Mean SIR for each of the lower concentration treatment groups (mean ± SEM). All three compounds reduced the extent of 2D migration, but Turmeric (0.032 µg/mL) exerted the most statistically significant effect; *n* ≥ 11 for each drug treatment. ANOVA: * = *p* ≤ 0.05, and *** = *p* ≤ 0.001.

Each of the three plant-derived drugs significantly reduced the extent of migration into scratches, with Turmeric exerting the most effective anti-migratory effect in 2D. After 24 h, the initial scratch edges were less distinct in the control, Magnolia (0.116 µg/mL) and Indigo (0.027 µg/mL) treatment groups (Figure 5a). In contrast, Turmeric (0.032 µg/mL) treatment visually reduced the migration into scratches the most, with a high cell confluency visible at the scratch edges after incubating for 24 h, unlike any of the other treatment groups.

The quantification of 2D migration via SIR determination demonstrated that treatment with Turmeric (0.032 µg/mL) significantly decreased migration the most, although both Magnolia (0.116 µg/mL) and Indigo (0.027 µg/mL) displayed migrastatic activity in U87 cells (Figure 5b). It is worth noting that none of the three plant-derived drugs completely inhibited migration.

#### 3.3.2. Drugs Administered at a High Concentration

**Figure 6:**
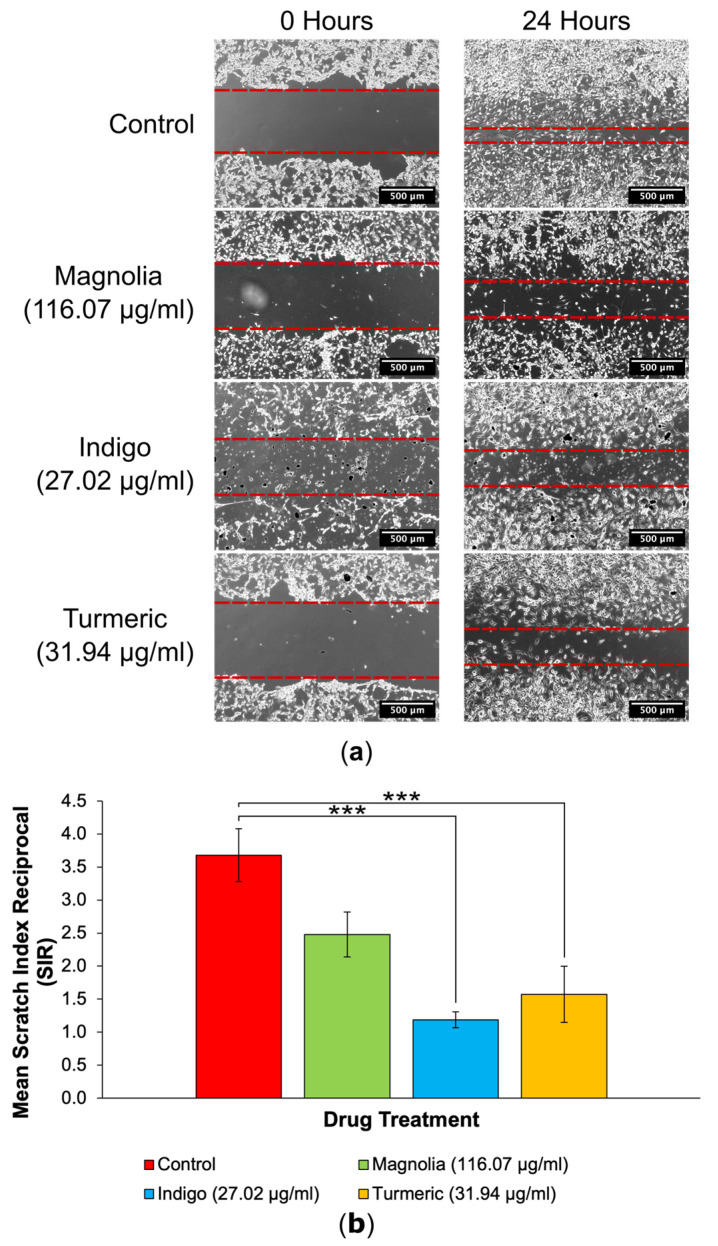
Two-dimensional migration into scratches is significantly reduced by plant-derived compounds with established anti-cancer activity at their IC_25_ concentrations. (**a**) EVOS XL Core generated images demonstrated the extent of migration into scratches after 24 h incubation. Dashed red lines are used to highlight the wound edges. Scale bar = 500 µm. (**b**) Mean SIR for each of the lower concentration treatment groups (mean ± SEM). All three compounds reduced 2D migration, but Indigo (27.02 µg/mL) produced the lowest SIR, although this was just as statistically significant as for Turmeric when compared to the control; *n* ≥ 11 for each drug treatment. ANOVA: *** = *p* ≤ 0.001.

The images acquired of the scratches following 24 h incubation demonstrated that at the higher plant-derived drug concentrations, the effect on cell migration was more pronounced (Figure 6a). A visible cell front was distinguishable in scratches treated with each of the three compounds. All three treatment groups reduced the migration of U87 cells into scratches in comparison to the control treatment; however, Magnolia (116.07 µg/mL) and Indigo (27.02 µg/mL) appeared to exert an enhanced inhibitory effect on 2D glioma cell migration.

Interestingly, treatment with Indigo (27.02 µg/mL) had the lowest SIR compared to the control group, although this was equally as statistically significant as for Turmeric (31.94 µg/mL) (*p* < 0.001), indicating that both Indigo and Turmeric exert 2D anti-migratory activity on U87 cells (Figure 6b). Magnolia (116.07 µg/mL) also reduced the mean SIR, but this was not as significant as for Indigo or Turmeric. Therefore, all three compounds are capable of reducing glioma cell migration in a 2D environment, but do not completely inhibit migration.

### 3.4. Spheroid Invasion Assays

All drugs were assessed for their activity on migration/invasion in a 3D environment in the spheroid invasion assays. Two MIs were established, the migration front and migration edge. This allowed further investigation of drug activity in cell populations, i.e., the bulk cell population migrating away from the spheroid, and single, highly migratory cells ahead of the main bulk of migrating cells. MIs for both the front and edge were 0 at 0 h for all spheroids as no cells had migrated away from the original spheroids straight after embedding in collagen.

#### 3.4.1. Drugs Administered at a Low Concentration

**Figure 7:**
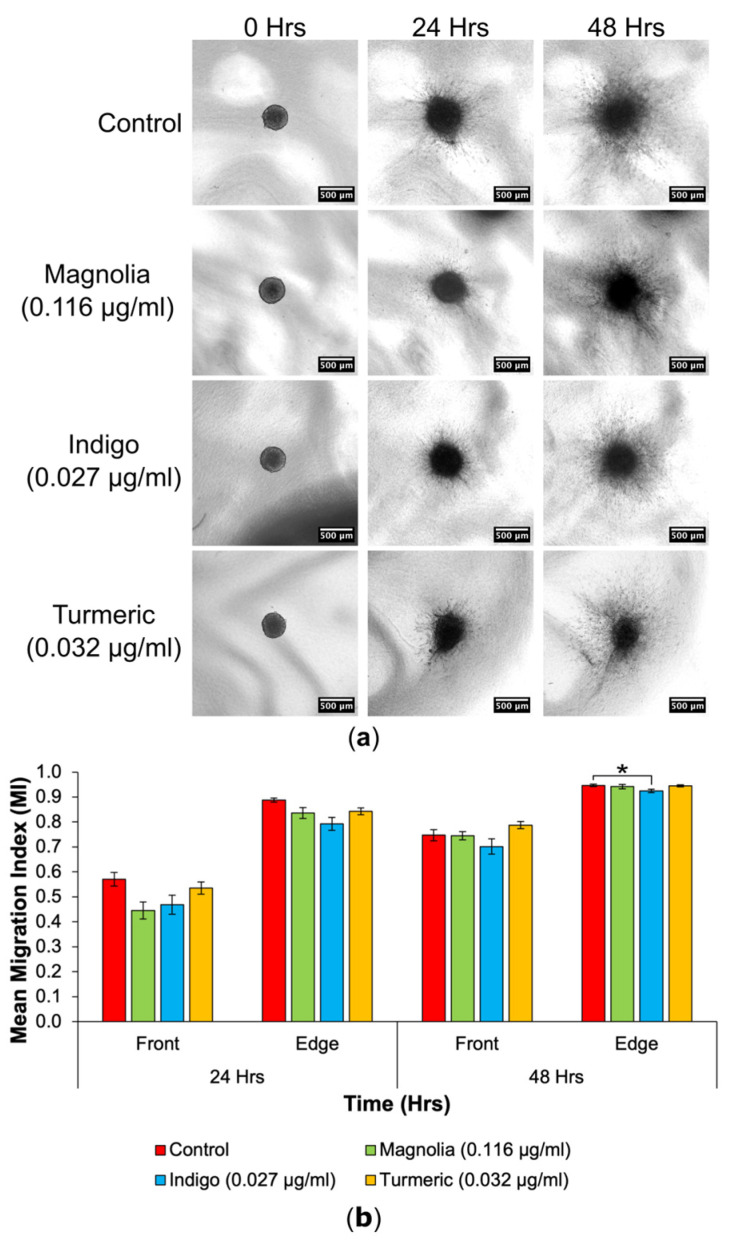
Cell migration from spheroids was reduced following treatment with a low concentration of natural products. (**a**) Images obtained using an EVOS XL Core imaging system showed an increase in migration into the collagen matrix over a 48 h period. Spheroids in the treated groups appeared visually consistent with the control. Scale bar = 500 µm. (**b**) Mean MIs for each of the lower drug concentrations, at the migrating front and edge at all timepoints (mean ± SEM). Indigo (0.027 g/mL) treated spheroids had the most significantly reduced edge MI at 48 h. Magnolia (0.116 µg/mL) exerted the most anti-migratory effect for the migrating front after 24 h, but this was not statistically significant; *n* ≥ 7 for each drug treatment. ANOVA (front MI 24 h, edge MI 24 h and front MI 48 h) and Kruskal-Wallis (edge MI 48 h): * = *p* ≤ 0.05.

Three-dimensional cell migration into the collagen matrix appeared to be reduced in the Turmeric (0.032 µg/mL) treated group and migrating cells seemed less densely populated around the spheroid in comparison to the control (Figure 7a). In addition, Turmeric-treated spheroids seemed to change the least in size, as spheroids from all other treatment groups increased over the 48 h incubation.

At 24 h, spheroids treated with Magnolia (0.116 µg/mL) exhibited the biggest reduction in cell migration, and Indigo treatment produced the lowest edge MI, but there were no significant differences determined at 24 h (Figure 7b). Indigo had the most inhibitory effect on cell migration at the migrating front after 48 h, whilst Turmeric treatment appeared to increase the mean front MI, although any differences between the control and drug treatments were insignificant. For the migrating edge at 48 h, Indigo induced the biggest reduction in migration, and was the only statistically significant result observed. Although Turmeric (0.032 µg/mL) displayed the most migrastatic activity in 2D, this was not recapitulated in 3D. Again, the three plant-based drugs failed to completely halt migration.

#### 3.4.2. Drugs Administered at a High Concentration

**Figure 8:**
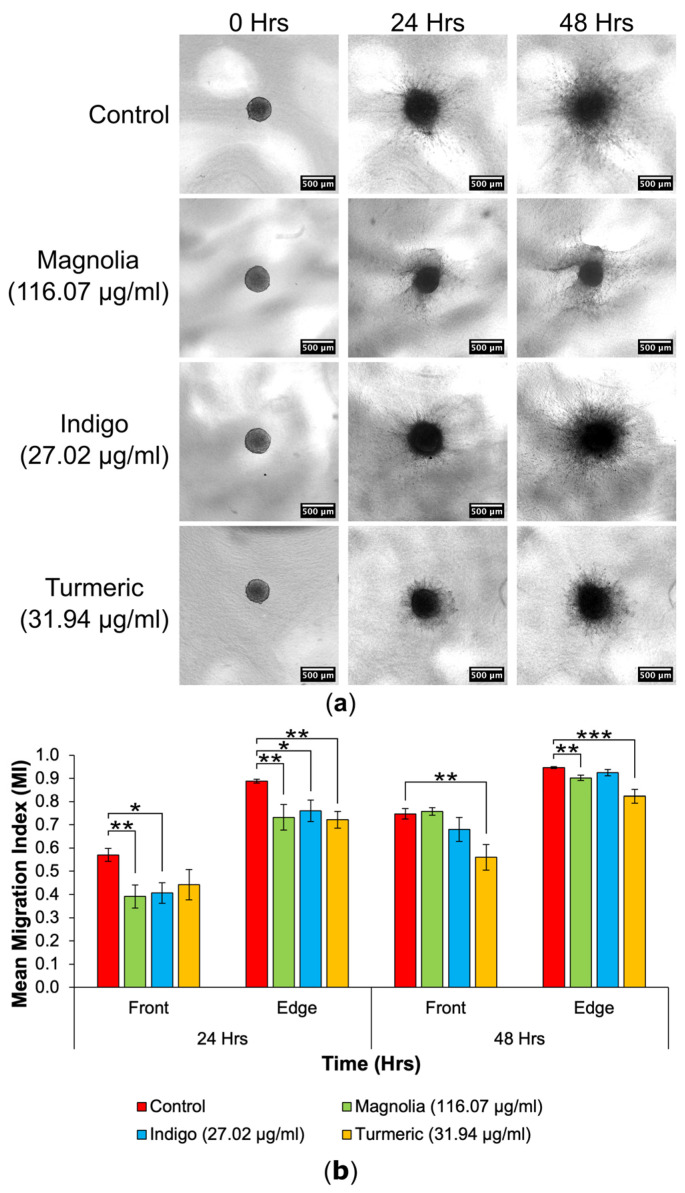
Decrease in 3D GBM cell migration after treatment with a high concentration of plant-based drugs. (**a**) Spheroid images taken over 48 h highlight a decrease in migration in the Turmeric (31.94 µg/mL) group. Scale bar = 500 µm. (**b**) The mean MI of both treated and mock-treated spheroids (mean ± SEM). At the 24 h migrating edge and both 48 h fronts, Turmeric (31.94 µg/mL) produced the lowest and most statistically significant MI; *n* ≥ 7 for each drug treatment. ANOVA (front MI 24 h, edge MI 24 h and front MI 48 h) and Kruskal-Wallis (edge MI 48 h): * = *p* ≤ 0.05, ** = *p* ≤ 0.01 and *** = *p* ≤ 0.001.

Of all the drug treatments, Turmeric (31.94 µg/mL) appeared to reduce migration into the collagen matrix the most at both 24 and 48 h, with migrating cells remaining closely associated with the original spheroid (Figure 8a).

For the migrating front at 24 h, Magnolia (116.07 µg/mL) and Indigo (27.02 µg/mL) treatment significantly reduced the mean MI, with Magnolia exhibiting the most inhibitory effect (Figure 8b). All three drugs decreased the mean edge MI after 24 h incubation, but Turmeric had the highest anti-migratory effect (*p* < 0.01), although all three treatments were statistically significant when compared to the control. However, at 48 h, Turmeric-treated spheroids exhibited the lowest mean MI for both the migrating front and edge. The effect of Turmeric was enhanced for the migrating front compared to the edge. Cell migration continued from the spheroids into the collagen matrix, despite treatment with each of the three compounds.

#### 3.4.3. Spheroid Confocal Microscopy

**Figure 9:**
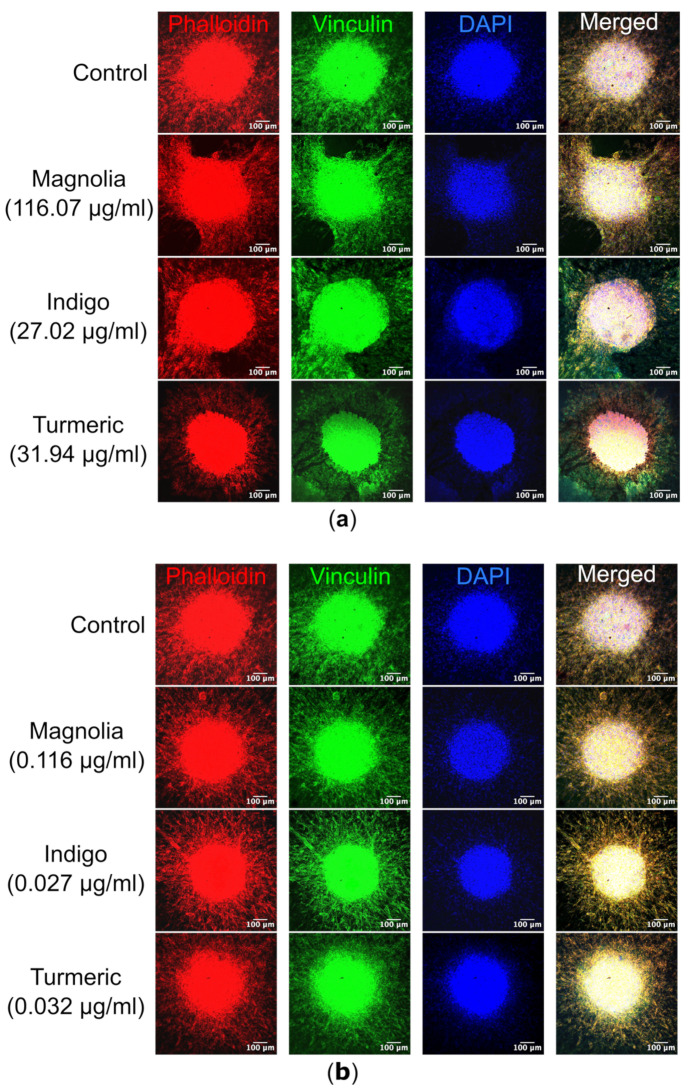
Immunofluorescence of both migrating U87 cells and spheroids within their respective collagen plugs. (**a**) Images of each treatment group were acquired, with Alexa fluor 488 (secondary to mouse anti-vinculin) excited at a wavelength of 488nm, TRITC-conjugated phalloidin at 561 nm and DAPI at 405 nm. Turmeric (31.94 µg/mL) cells are morphologically rounded in comparison to the control, and actin is less visible within migrating cells. Scale bar = 100 µm. (**b**) Similarly, spheroids treated with the lower concentrations of the plant-based compounds were visualized, but there were no obvious differences in cell morphology as a result of drug treatment. Red = actin, green = vinculin and blue = DAPI. *n* ≥ 2 for each drug treatment. Scale bar = 100 µm.

The visualization of spheroids and migrating cells in their collagen plugs via confocal microscopy highlighted the differences in cell morphology as a result of drug treatment. The most noticeable effect was observed after treatment with Turmeric (31.94 µg/mL), with migrating cells possessing a rounded morphology, compared to the elongated cell body observed in the control mock-treated spheroids (Figure 9a). In Turmeric (31.94 µg/mL) treated spheroids, actin appeared concentrated in the spheroid core. However, this effect was not observed with the lower Turmeric concentration (0.032 µg/mL), suggesting that the effects on migration are concentration dependent (Figure 9b). Both Turmeric (31.94 µg/mL) and Indigo (27.02 µg/mL) treatments appeared to result in sheet-like migration, rather than the single-cell motility seen in the control. For Magnolia and Indigo, at both low and high concentrations, the cell morphology was consistent with the control.

## 4. Discussion

Treatment options for GBM remain unchanged and mainly focus on exerting cytotoxicity, although these treatments are failing to offer a significant survival benefit to patients. No current GBM treatment functions to target cell migration, despite high migratory activity in GBMs being directly associated with invasion into healthy brain parenchyma and therefore tumor recurrence, highlighting that this is an area of unmet need in GBM management. Hence, there is an urgent demand for novel or repurposed treatment options to improve GBM patient survival by targeting migration in combination with standard treatments like surgery, chemotherapy and radiotherapy. Plant-based compounds are of particular interest in GBM research as they have been shown to exhibit anti-cancer activity with associated low, non-specific cytotoxic side effects; however, any potential effects on cell migration are less well documented. Therefore, we investigated the anti-migratory activity of three plant-derived compounds with known cytotoxicity, Turmeric, Indigo and Magnolia Bark, to determine if they could be potentially used in combination with standard GBM treatments to promote patient survival. Our study found that the three compounds tested exhibited anti-migratory effects at higher concentrations, with Turmeric exerting a pronounced and novel effect on FA dynamics, and migration in both 2D and 3D environments. The effects on glioma cell migration were also observed following treatment with Indigo and Magnolia; however, these were much less pronounced than for Turmeric.

### 4.1. Turmeric

Microscopic examination at the subcellular level revealed changes in actin localization and FA dynamics following treatment with the plant-derived compounds, as hypothesized, as both proteins are required to promote cell adhesion, structure and movement [29]. Decreased numbers of FAs were noted in the Turmeric group, concomitant with a striking rounded, amoeboid morphological phenotype. A distinct absence of actin stress fibers in Turmeric-treated cells suggests that Curcumin can induce a mesenchymal amoeboid transition (MAT), as amoeboid cells require peripheral actin to generate the constant polarity changes necessary for migration [30]. MAT has been previously documented in glioma cells, reducing Rac1 activity whilst increasing ROCK expression, and our previous studies have also highlighted phenotypic switching in glioma cells [31,32,33,34]. Cytoskeletal changes observed following treatment with Turmeric have been previously noted, where cell migration inhibition occurred through actin and FA regulation, as previously demonstrated [35]. Cofilin, a protein which functions to either extend or shorten actin filaments, was detected at reduced levels as a result of Turmeric treatment, suggesting a potential role of Curcumin in targeting the cytoskeleton [36,37]. It has been hypothesized that the effect of Turmeric on FA generation occurs via FAK (focal adhesion kinase), allowing regulatory control of cell migration [38]. Previous studies have also shown that Curcumin reduces fascin expression, resulting in decreased filopodia formation in U87 cells [39]. To our knowledge, our observation of the effect of Turmeric on FA dynamics in glioma cells has so far not been reported.

In line with our findings, previous studies carried out also highlighted the anti-migratory activity of Curcumin using non-small cell lung cancer cells [40]. It is interesting that the effects on the cells migrating the furthest away from the spheroid (indicated by the migration edge) for Turmeric-treated cells were more significant than those on the migrating cell bulk front; we suggest that Curcumin may be particularly targeting highly migratory single GBM cells likely to acquire treatment resistance and contribute to tumor recurrence [41]. Despite Turmeric’s ability to significantly reduce the migration of cells at the front and edge, migration was not completely inhibited, suggesting a switch to an alternative signaling pathway and form of motility. The visualization of the spheroids and migratory cells in their collagen plugs via confocal microscopy supports our suggestion of a morphological transition to amoeboid cell morphology and cell migration/invasion induced by Turmeric, compared to the elongated cell bodies observed in cells emanating from the control, mock-treated spheroids, although this effect was not observed at the lower concentration. In addition, we suggest a possible switch to collective cell migration in response to Turmeric, which has been noted in established glioma cell lines following anti-migratory drug treatment [34].

**Figure 10:**
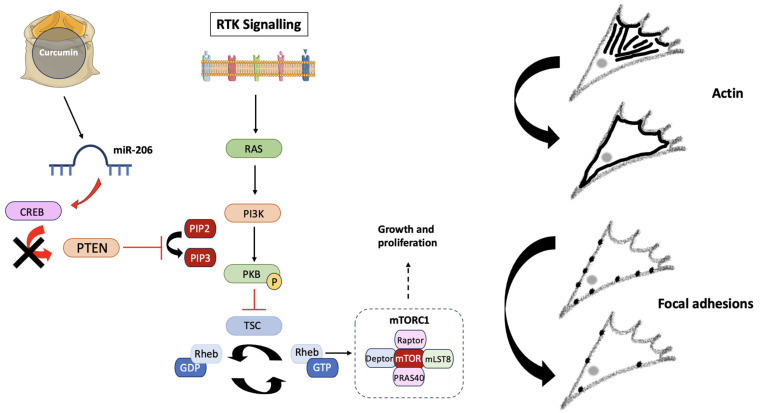
The potential effects of Curcumin on the PI3K-PKB-mTOR pathway. Receptor tyrosine kinases (RTKs) activate Ras and PI3K following the conversion of PIP2 into PIP3. PKB is then activated via phosphorylation, where it inhibits tuberous sclerosis protein (TSC), a GTPase responsible for converting Rheb-GTP into Rheb-GDP. Rheb (Ras homolog enriched in brain) controls mTORC1 expression, exerting effects on growth and proliferation. Curcumin, the active compound of Turmeric, has been shown to up-regulate miR-206, which reduces CREB protein expression, preventing PTEN down-regulation and therefore inhibiting mTORC1 activity by preventing PKB phosphorylation. The lack of mTORC1 activity induces changes in cytoskeletal dynamics, due to direct effects on migration. Created using Smart Servier Medical Art (https://smart.servier.com/ (accessed on 3 May 2024)).

GBM cell migration is a complex process governed by many cellular mechanisms and pathways, many of which become dysregulated via acquired mutations to oncogenes and tumor suppressor genes (TSGs) [42]. As a product of a TSG, the phosphatase and tensin homolog (PTEN) protein acts to regulate several signaling pathways involved in cell growth and division [43] (Figure 10). Mutations resulting in a loss of function to *PTEN* promote increased proliferative activity, giving rise to additional mutations which could potentially result in GBM cell survival [44]. The PI3K-PKB/Akt-mTOR pathway is one such signaling mechanism under the regulatory control of PTEN, with roles in cell survival and proliferation [45]. Once the proto-oncogene Ras is activated upon receptor tyrosine kinase binding in healthy cells, the PI3K (phosphoinositide-3-kinase) signaling cascade is subsequently triggered [46]. Resulting in the activation of second messengers, PI3K initiates mammalian targeting of rapamycin complex 1 (mTORC1) expression, to stimulate proliferation. Protein kinase B (PKB) activation also occurs via a PI3K-mediated mechanism; however, this can be halted by PTEN [47].

Research has suggested that Curcumin exerts its effects on non-small cell lung cancer migration due to the inhibition of the PI3K-PKB-mTOR signaling pathway, which acts to increase miR-206 expression and ultimately prevent carcinogenesis [40]. By decreasing CREB (cyclic-AMP response binding element) expression, miR-206 can act as an activator or inhibitor of tumor progression. In the case of GBMs, CREB activity exerts control over *PTEN*, promoting increased proliferation [48]. The anti-migratory effects of Turmeric can potentially be attributed to the suppression of the PI3K-PKB-mTOR pathway and miR-206 overexpression, consequently increasing PTEN expression due to decreased CREB activation. Treatment strategies targeting the PI3K-PKB-mTOR pathway result in reduced chemotherapy resistance and improved patient outcomes, implying that the use of Turmeric in targeting this signaling pathway is clinically relevant [49,50].

Cell migration often utilizes a leader-follower system, with cells gaining a benefit over the surrounding cells to essentially carve a path for the follower cells to take advantage of [51]. Little is known about the molecular and cellular differences between follower and leader cells; however, the ability of Turmeric to target GBM leader cells demonstrates its therapeutic potential, as the leader or edge cells directly contribute to GBM recurrence and patient death [52].

### 4.2. Indigo

Indirubins are a group of active compounds found in Indigo which exert cytotoxic effects [53]. BIO is part of the Indirubin group and has demonstrated efficient migrastatic activity in reducing GBM cell migration through a process of GSK-3 inhibition, therefore resulting in cytoskeletal changes and reduced FA generation [25]. As Indigo only induced a significant decrease in cell migration from spheroids after 24 h, it is suggested that the compound degrades quickly. Therapeutic compounds are required to remain at a constant concentration within the brain during treatment, so the short duration of Indigo potentially limits its translation into the clinic and suggests the need for a carefully planned treatment regime over a defined time period [54]. The effect of Indigo on migration from spheroids was more pronounced at the migrating front at both concentrations and all timepoints, indicating that its anti-migratory effect is much less pronounced in GBM leader cells, potentially limiting the use of Indigo in patients.

**Figure 11:**
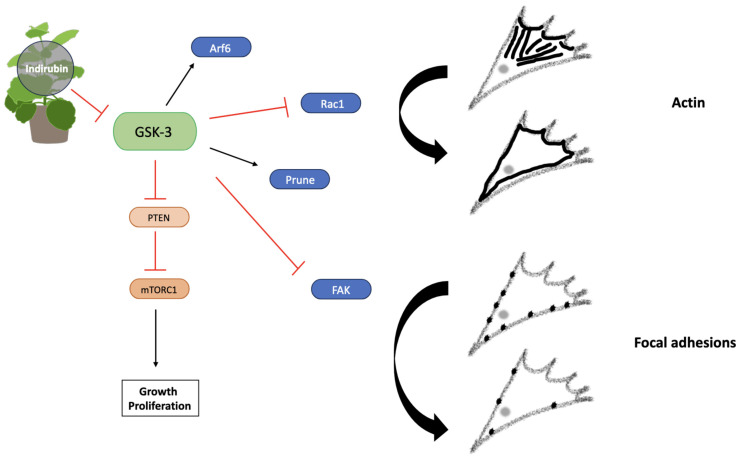
Indirubin exerts anti-migratory effects via GSK-3 inhibition. Arf6 activation induced by GSK-3 promotes actin localization, whilst Prune increases FAK expression. Maintenance of FA turnover is controlled by GSK-3-mediated FAK inhibition. PTEN inhibition also depends on GSK-3 expression, promoting activation of the PI3K-PKB-mTORC1 pathway, therefore leading to growth, proliferation and migration. Actin localization and FA dynamics are altered via GSK-3 inhibition. Created using Smart Servier Medical Art (https://smart.servier.com/ (accessed on 3 May 2024)).

Research has highlighted the presence of cortical actin localization and amoeboid morphology following GSK-3 inhibition, suggesting that Indigo is capable of targeting several signaling pathways or that different derivatives exert distinct effects on cell morphology [55] (Figure 11). GSK-3 functions in several signaling pathways involved in cell migration such as PTEN suppression, actin expression via Rac activation and FAK inhibition, changing the cytoskeletal dynamics to ultimately alter motility [56]. It is worth noting that cells migrating from spheroids in the higher concentration group appeared to move in a sheet-like manner indicative of collective cell migration.

### 4.3. Magnolia

**Figure 12:**
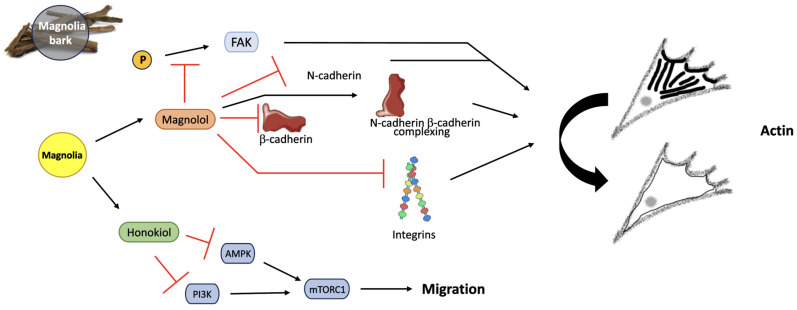
Magnolia bark reduces cell migration through the action of both APIs, Magnolol and Honokiol. FAK phosphorylation is inhibited by Magnolol, down-regulating N-cadherins, β-cadherins and integrins. N-cadherin and β-cadherin complexation is increased by Magnolol, resulting in reduced migration. AMPK and PI3K are inhibited by Honokiol, consequently decreasing mTORC1 activity and migration by altering actin localization in the cytoskeleton. Created using Smart Servier Medical Art (https://smart.servier.com/ (accessed on 3 May 2024)); Magnolia bark image created by siewlingc (https://www.freeimg.net/photo/1000145/traditionalchinesemedicine-chineseherb-bark (accessed on 3 May 2024)).

As one of Magnolia’s active agents, the efficiency of Magnolol as a migrastatic has been proposed to occur as a result of reduced FA expression, membrane-bound N-cadherin expression and a lack of FAK phosphorylation [57] (Figure 12). N-cadherins (neural-cadherins) are overexpressed when cells gain the mesenchymal features required to invade surrounding tissues as a unit [58,59]. The impact of cadherin expression on glioma survival has been questioned, however [60]. Magnolia’s second active compound, Honokiol, has exhibited both anti-migratory and cytotoxic activity on cancer cells, with autophagy being triggered at high concentrations due to decreased PI3K activity, therefore inhibiting the functions carried out by the PI3K-PKB-mTOR pathway [61]. It is believed that reduced mTOR activity occurs due to AMPK (5′ AMP-activated protein kinase) expression, consequently inhibiting cell migration [62]. Although this mechanism is not fully understood, Magnolia may act via similar signaling pathways to those of Turmeric. Magnolia treatment failed to completely halt GBM cell migration in all assays carried out, perhaps suggesting a switch to an alternative method of movement; therefore, further research is required to determine if cells possess the capacity to acquire treatment resistance after exposure to each of the plant-based drugs.

## 5. Conclusions

In this study, 2D and 3D migration and invasion assays have demonstrated the anti-migratory activity of three plant-derived compounds with previously established cytotoxic, anti-cancer activity. We report that Turmeric, Magnolia and Indigo all show promise as migrastatic drugs, with Turmeric exhibiting the most pronounced effect on migration, potentially via a PTEN-mediated mechanism. A significant decrease in GBM cell migration, particularly at the spheroid migrating edge, was observed following treatment with Turmeric, emphasizing the potential role of Curcumin as a GBM therapeutic to prevent tumor recurrence and ultimately improve patient survival.

The cytotoxic activity of plant-derived compounds on cancer cells has been frequently reported in the literature, making them molecules of great interest. However, the repurposing of these compounds as migrastatics is less well documented and is a novel approach to prevent tumor recurrence and improve patient survival. This study highlights that the repurposing of naturally sourced compounds with proven cytotoxic and anti-cancer activity is a cost-effective and efficient strategy to target cell migration in highly motile cancer cells such as GBMs. As our approach to use plant-derived products as migrastatics has demonstrated Turmeric’s anti-migratory activity, further investigation should be carried out to elucidate the signaling pathways involved. In addition, we will continue our studies using patient-derived cell lines to confirm if our observed effects can be reproduced in a more clinically relevant background. Turmeric is readily available and inexpensive, with no discernible non-specific side effects; therefore, future studies should also determine the effects of a combinatory approach with the standard cytotoxic treatment agent, Temozolomide (TMZ), to provide improved treatment options for brain tumor patients.

## Data Availability

Data will be made available upon request.

## Supplementary Materials

The following supporting information can be downloaded at: https://www.mdpi.com/article/10.3390/jox14020036/s1. Figure S1: MTT assay results for the three compounds tested, Magnolia Bark, Turmeric and Indigo to determine the concentration at which at least 75% cell viability was observed. Various concentrations of the compounds were added in duplicate to the wells. The assay was repeated twice. The results are expressed as percentage cell viability.
