# Supplementary material for: Traditional Plant-Derived Compounds Inhibit Cell Migration and Induce Novel Cytoskeletal Effects in Glioblastoma Cells
Source: J Xenobiot. 2024 May 9;14(2):613–33. doi: 10.3390/jox14020036 (PMC11130960; doi:10.3390/jox14020036)
Supplement: Supplementary file 1 [file jox-14-00036-s001.zip › jox-2928266-supplementary.pdf]

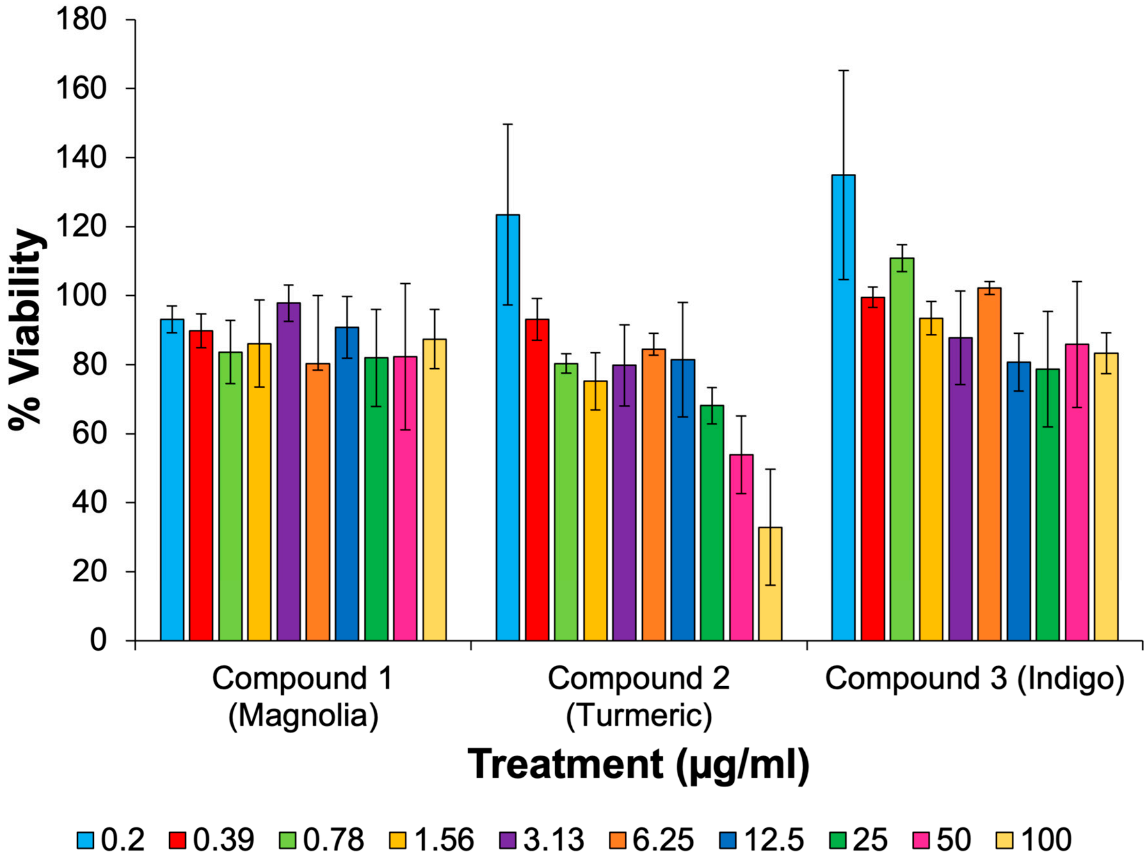

Figure S1. MTT assay results for the three compounds tested, Magnolia Bark, Turmeric and Indigo to determine the concentration at which at least 75% cell viability was observed. Various concentrations of the compounds were added in duplicate to the wells. The assay was repeated twice. The results are expressed as percentage cell viability.
